# Communication in radiology: evaluation of terminology and TNM descriptor use at a cancer center

**DOI:** 10.1590/0100-3984.2022.0043

**Published:** 2022

**Authors:** Thiago Pereira Fernandes da Silva, Gustavo Gomes Mendes, Valdair Francisco Muglia, Rubens Chojniak, Paula Nicole Vieira Pinto Barbosa

**Affiliations:** 1 A.C. Camargo Cancer Center, São Paulo, SP, Brazil.; 2 Faculdade de Medicina de Ribeirão Preto da Universidade de São Paulo (FMRP-USP), Ribeirão Preto, SP, Brazil.

**Keywords:** Patient-centered care, Radiology/standards, Medical records, Interprofessional relations, Communication barriers, Terminology as topic, Assistência centrada no paciente, Radiologia/normas, Registros médicos, Relações interprofissionais, Barreiras de comunicação, Terminologia como assunto

## Abstract

**Objective:**

The purpose of our study was to evaluate the transmission of information from radiologists to physicians, focusing on the level of certainty and the use of imaging descriptors from the tumor–node–metastasis (TNM) staging system.

**Materials and Methods:**

Radiologists (n = 56) and referring physicians (n = 50) participated in this questionnaire-based, singlecenter study, conducted between March 20, 2020, and January 21, 2021. Participants were presented with terms commonly used by the radiologists at the institution and were asked to order them hierarchically in terms of the level of certainty they communicate regarding a diagnosis, using a scale ranging from 1 (most contrary to) to 10 (most favoring). They then assessed TNM system descriptors and their interpretation. Student’s t-tests and the kappa statistic were used in order to compare the rankings of the terms of certainty. Items related to T and N staging were analyzed by Fisher’s exact test. The confidence level was set to 97% (*p* < 0.03).

**Results:**

Although overall agreement among the radiologists and referring physicians on term ranking was poor (kappa = 0.10– 0.35), the mean and median values for the two groups were similar. Most of the radiologists and referring physicians (67% and 86%, respectively) approved of the proposal to establish a standard lexicon. Such a lexicon, based on the participant responses, was developed and graphically represented. Regarding the TNM system descriptors, there were significant differences between the two groups in the reporting of lymph node numbers, of features indicating capsular rupture, and of vessel wall irregularities, as well as in the preference for clear descriptions of vascular involvement.

**Conclusion:**

Our findings indicate that ineffective communication and differences in report interpretation between radiologists and referring physicians are still prevalent in the fields of radiology and oncology. Efforts to gain a better understanding of those impediments might improve the objectivity of reporting and the quality of care.

## Abstract (pt)

**Objetivo:**

O propósito do nosso estudo foi analisar a transmissão das informações de radiologistas para médicos assistentes, com foco no nível de certeza e uso de descritores de imagem do sistema de estadiamento tumor–nódulo–metástase (TNM).

**Materiais e Métodos:**

Radiologistas (n = 56) e médicos assistentes (n = 50) participaram neste estudo unicêntrico, baseado em questionários respondidos entre 20 de março de 2020 e 21 de janeiro de 2021. Os participantes ordenaram hierarquicamente termos comumente usados por radiologistas da instituição para descrever o nível de certeza utilizando uma escala que variou de 1 (mais contrário) a 10 (mais favorável). Em seguida, foram avaliados os descritores relacionados ao sistema TNM e sua interpretação. O teste t de Student e o coeficiente de correlação kappa foram empregados para comparar a classificação dos termos. Os itens relacionados aos estadiamentos T e N foram analisados pelo teste exato de Fisher. O nível de confiança foi fixado em 97% (*p* < 0,03).

**Resultados:**

A concordância geral entre radiologistas e médicos assistentes na classificação hierárquica dos termos foi baixa (kappa = 0,10–0,35), porém, os valores médios e medianos dos dois grupos foram semelhantes. A maioria dos médicos (86%) e radiologistas (67%) foi receptiva à introdução de um léxico padronizado. Uma proposta de léxico foi elaborada com base nas respostas dos participantes e representada graficamente. Em relação aos descritores do sistema TNM, diferenças estatisticamente significativas foram observadas nos seguintes itens: forma de relatar o número de linfonodos; menção a características indicativas de ruptura capsular nodal; menção de irregularidades nas paredes vasculares; e preferência por descrições sucintas para comprometimento de estruturas vasculares.

**Conclusão:**

Falhas de comunicação e diferenças na interpretação de laudos entre radiologistas e médicos assistentes ainda são prevalentes em radiologia e oncologia. Esforços para melhor compreendê-los podem melhorar a objetividade do laudo radiológico e a qualidade do atendimento médico.

## INTRODUCTION

The importance of communication in diagnostic radiology is recognized in the literature^(1,2,3)^. Siewert et al.^(1)^ showed that up to 38% of communication errors directly interfered with patient care. Economic issues are also noteworthy; Brenner et al.^(2)^ found that compensation costs in situations involving miscommunication can be up to twice as high as those incurred when appropriate communication is established.

Diagnostic possibilities are often raised based on imaging findings in radiology practice. Between descriptions of pathognomonic findings (e.g., a tibial shaft fracture) and the absence of a finding (e.g., lack of pneumothorax), there is a spectrum of terms employed to represent the level of certainty for a diagnostic hypothesis. Reviews of the literature have shown that the application of a wide variety of terms according to individual preference can lead to divergent interpretations^(1,4)^. Khorasani et al.^(4)^ found poor agreement among radiologists and referring physicians on the ranking of 15 terms to express probability in radiology reports, from most to least certain. An initiative at the Memorial Sloan Kettering Cancer Center to narrow the terms employed to denote the level of certainty and to create a standard lexicon, implemented gradually over five years and requiring cultural adaptation, ultimately resulted in broad approval of and adherence to the lexicon^(4)^. Therefore, such efforts to improve the communication of radiological impressions, although difficult and demanding, are worthwhile and justified.

### Communication in oncological radiology

The cancer burden increases annually, with an estimated economic impact of nearly 1 trillion dollars worldwide**(^5^).** Developed in France in the 1940s by Pierre Denoix, the tumor–node–metastasis (TNM) international oncological classification system has become the most widely accepted and used system for tumor staging. The recommendations are periodically updated, based on the latest scientific evidence, by the Union for International Cancer Control (UICC) and the American Joint Committee on Cancer (AJCC). The TNM system categories are specific to tumor type and were established based on prognostic studies and the experience of experts in different fields^(6)^.

The correct use of the TNM system results in better patient care. Nevertheless, its application by radiologists remains a challenge. Ko et al.^(7)^ stated that no Englishlanguage study has assessed whether radiology reports provide all of the information considered to be clinically relevant for accurate staging. They stated that structured reports based on the AJCC/UICC criteria are a reporting option that is as complete as possible to guide the therapeutic decisions of referring physicians. For head and neck cancer, they found that although most radiologists considered the TNM system to be a standard to be followed, only 24% used it routinely in written reports^(7)^.

The TNM system, albeit well formulated, does not encompass descriptions of the relevance of all radiological features to tumor staging. Several imaging aspects observed in practice do not have reliable references in this system, and their description (or lack thereof) depends on the personal experience and individual interpretations of radiologists, as well as on institutional practice and policy. For example, pertinent nodal involvement features differ among cancer types, and some of those features are hardly addressed in the TNM system manual^(6)^. In addition, several descriptors, even the most traditional ones (e.g., the cutoff point of 10 mm on the shortest axis for lymph node measurement), lack sufficient evidence of reliability and are therefore presented with reservation or not considered.

In the “T” category of the TNM system, primary tumor measurement has been universally adopted. Although the AJCC cancer staging manual^(6)^ recommends measurement of the largest diameter for most cancers, measurements taken in two or three orthogonal planes are often reported. For reporting on anatomical relationships, various expressions— such as “intimate contact”, “direct contact”, “no cleavage plane”, and “no unequivocal sign of invasion (of adjacent structures)”—may be applied. Also noteworthy are vessel wall irregularities (stenosis, with or without caliber reduction due to extrinsic compression), vascular flow patency, and radial contact with the vessel (e.g., “90° contact with the superior mesenteric artery”). In the “N” category, radiologists report node measurements and counts, and describe other radiological features—such as morphology, central hypodensity (resembling necrosis), contour abnormalities, and capsular rupture signs—according to their practice and experience. The “M” category, representing the probability that a lesion corresponds to distant metastasis, is closely related to the radiological impression. Radiologists report their degree of suspicion for lesions based on the consistency between imaging findings and the clinical and epidemiological behavior of given neoplasms.

Referring physician interpretation of descriptions of radiological staging features in oncologic imaging reports is another important issue. An initiative that addresses this topic might be opportune, because the identification and avoidance of confounding factors could improve the quality of care. In this context, this questionnaire-based study was conducted to evaluate the transmission of information about the level of certainty and imaging descriptors from the TNM staging system from radiologists to referring physicians.

## MATERIALS AND METHODS

### Study design and sample

This was a cross-sectional, single-center, anonymous, prospective study conducted between March 2020 and January 2021. A total of 106 physicians (56 radiologists and 50 referring physicians) participated. The data obtained were stored in a restricted-access database. We included only fully completed questionnaires duly submitted by collaborators of the center. The medical specialties of the 50 attending physicians were as follows: oncology (n = 13); surgical oncology (n = 7); radiotherapy (n = 6) pathology (n = 5); nuclear medicine (n = 5); anesthesiology (n = 4); dermatology (n = 2); thoracic surgery (n = 2); urology (n = 2); infectology (n = 1); plastic surgery (n = 1); pediatric oncology (n = 1); and orthopedics (n = 1). The study was approved by the local research ethics committee, and all of the participants provided written informed consent.

### Data collection

Data were collected prospectively using a two-part questionnaire developed by the authors, who were resident or senior oncologic imaging staff members, and was carried out through the use of the online GoogleForms and RedCap platforms. Participants were informed of the goals of the study beforehand. In the first part of the questionnaire, we used a variation of the method proposed by Khorasani et al.^(4)^ to assess concordance on terms related to certainty. Participants were presented with ten terms related to the level of certainty for grading that were commonly used by radiologists in their reports at the institution. Each participant was asked to order the terms hierarchically on a scale ranging from 1 (most contrary to a diagnostic possibility) to 10 (most favoring a diagnostic possibility) and to select five terms for inclusion in a graphic representation of a standard lexicon. Our main objective related to the first part of the questionnaire was to assess radiologist and referring physician ranking of and agreement with expressions commonly used for probability grading in radiology. The second part of the questionnaire addressed descriptors used routinely in oncologic imaging reports and their interpretation by referring physicians, based on the TNM system. Items were related to tumor measurement and the description of tumor contact with viscera and adjacent vessels, as well as the reporting of lymph node dimensions, number, and morphological features. The objective related to the second part of the questionnaire was to assess participant understanding of and agreement on TNM descriptors.

### Statistical analysis

To compare the ranking of terms of certainty between the two groups, parametric Student’s t-tests for independent samples were applied. The kappa statistic was employed to assess overall agreement between groups and measures of central tendency. Items related to T and N staging were analyzed using Fisher’s exact test to examine concordance and identify significant differences between groups. The confidence level was set to 97% (*p* < 0.03).

## RESULTS

**Figure 1:**
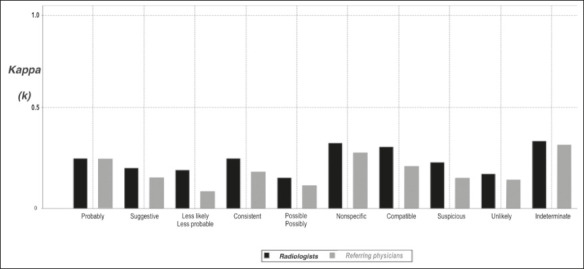
Agreement on the ranking of probabilistic terms. The low kappa values obtained in both groups (0.10–0.35) reflect a low degree of overall concordance.

**Figure 2:**
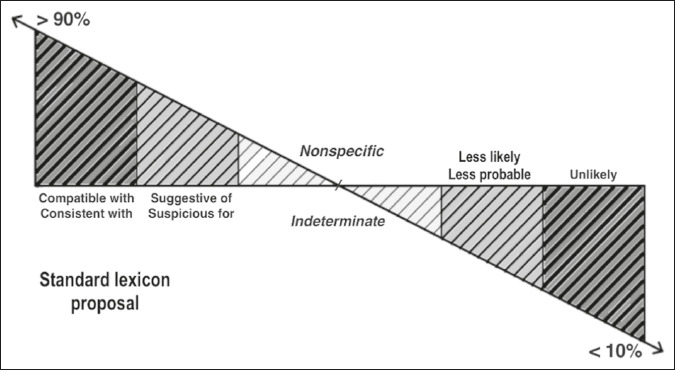
Proposed standard lexicon for the level of certainty about a diagnostic hypothesis, based on the top five terms selected by the participants. The graph represents a “convenience” variation of certainty ranging from < 10% to > 90%. Terms closer to 90% indicate a great degree of certainty of the part of a radiologist regarding the diagnostic hypothesis, those closer to 10% indicate greater conviction about refuting the diagnostic hypothesis, and those at the center of the graph indicate the most uncertainty about the diagnosis.

We obtained 106 completed questionnaires (56 from radiologists and 50 from referring physicians) between March 2020 and January 2021. Like Khorasani et al.^(4)^, we observed wide variability in the ranking of the probabilistic terms, as evidenced by the low kappa values (Figure 1). Measures of central tendency (mean, median, range, and standard deviation) are shown in Table 1. To minimize negative outcomes resulting from misinterpretation, we adopted the method of standard lexicon establishment suggested by Panicek et al.^(3)^. The lexicon constructed with the five terms selected by the most participants is presented in Figure 2. As shown in Table 2, most of the participants supported the proposed initiative to create a standard lexicon.

**Table 1:** Measures of central tendency for the ranking of terms commonly employed to convey the level of diagnostic probability in radiology reports.

Term	Radiologists				Referring physicians			
	Mean	Median	Range	SD	Mean	Median	Range	SD
Probably	7.75	8.00	3–10	1.083	8.03	8.00	6–9	0.797
Suggestive	7.80	8.00	5–9	0.961	7.59	7.50	5–10	1.131
Less likely/less probable	3.00	3.00	1–7	1.037	3.35	3.00	1–7	1.631
Consistent	9.00	9.00	3–10	1. 414	8.65	9.00	4–10	1.515
Possible/possibly	6.93	7.00	2–9	1.346	7.79	8.00	5–9	1.250
Nonspecific	4.48	5.00	1–10	1.839	4.59	5.00	1–10	2.363
Compatible	9.21	10.00	1–10	1.411	9.24	9.50	6–10	0.987
Suspicious	8.20	8.00	6–10	0.840	7.44	7.00	5–9	1.106
Unlikely	2.66	2.00	1–8	1.832	2.85	3.00	1–9	1.520
Indeterminate	4.54	5.00	1–10	1.868	4.91	5.00	1–9	1.975

SD, standard deviation.

**Table 2:** Overall acceptability of the proposal to create a standardized lexicon for the level of certainty.

Opinion	Total (N = 106) n (%)	Radiologists (N = 56) n (%)	Referring physicians (N = 50) n (%)	*P* -value*
Yes. I think it’s a great idea that can reduce misinterpretations in general.	81 (76)	38 (68)	43 (86)	0.0451
Maybe. I’m not totally convinced, but I would be willing to apply the lexicon whenever I think it’s plausible.	23 (22)	16 (28)	7 (14)	
No. I believe it will hinder/will not add value to my daily routine.	2 (2)	2 (4)	0 (0)	

* Fisher’s exact test.

The setting of a cancer center is ideal for the development of a study involving imaging descriptors from the TNM system. In that setting, we evaluated several of those descriptors related to the “T” and “N” category, as shown in Tables 3 and 4. We found statistically significant differences (*p* < 0.03) between the two groups in the preference for a brief description of vascular involvement, the reporting of vessel wall irregularities, the reporting of features indicating capsular rupture, and the reporting of lymph node numbers.

**Table 3:** Participant responses on items regarding T staging.

T-staging item	Radiologists (N = 56) n (%)	Referring physicians (N = 50) n (%)	*P* -value*
Primary tumor measurement			
One axis	10 (18)	9 (18)	
Two axes	24 (43)	22 (44)	1.000
Three axes	22 (39)	19 (38)	
Contact with adjacent viscera			
There is/isn’t a cleavage plane between the tumor and the anatomical structure	19 (34)	33 (66)	
There is/isn’t a clear fatty plane between the tumor and the anatomical structure	7 (12)	1 (2)	0.038
There is close contact between the lesion and the structure, without unequivocal signs of anatomical invasion	30 (54)	16 (32)	
Contact with vascular structures			< 0.001
Gives only a brief description: “Tumor extension to vessel _______ ”	35 (64)	47 (94)	0.377
Details circumferential involvement of the artery (e.g., “< 180°”)	50 (91)	42 (85)	0.081
Details the presence or absence of stenosis and alteration in vessel diameter	54 (96)	44 (88)	0.002
Details the presence or absence of vascular wall irregularities	43 (78)	24 (47)	0.768
Details the presence/absence of fow in the vessel	49 (89)	44 (88)	

* Fisher’s exact test.

**Table 4:** Participant responses on items regarding N staging.

N-staging item	Radiologists (N = 56) n (%)	Referring physicians (N = 50) n (%)	*P* -value*
Regional lymph nodes			
Measurement			
Major axis	5 (9)	10 (20)	
Minor axis	20 (36)	16 (32)	0.051
Two axes	30 (54)	17 (34)	
Three axes	1 (2)	7 (14)	
Numerical reporting			
Exact number	5 (9)	25 (50)	< 0.001
General idea of number (e.g., “few”, “multiple”)	45 (80)	24 (48)	
No mention of number	6 (11)	1 (2)	
Imaging descriptors			
Extracapsular rupture	33 (60)	46 (92)	< 0.001
Central density/signal changes (inferring necrosis)	53 (96)	43 (86)	0.082
Lymph node morphology (e.g., round, lobulated, or unusual)	52 (93)	39 (78)	0.056
Measurement	55 (98)	50 (100)	1.000
Precise anatomical terminology on lymph node chains	53 (96)	47 (94)	0.667

* Fisher’s exact test.

## DISCUSSION

### Level of certainty

Despite the wide variability in the ranking of the probabilistic terms, the analysis of measures of central tendency revealed that there was overall agreement between the two groups for many of the terms. For example, the mean ranking for “suggestive of” was 7.8 ± 0.96 (median, 8.0) among the radiologists and 7.59 ± 1.13 (median, 7.5) among the referring physicians, which are quite comparable values. That suggests that there was a common pattern for the quantification of certainty.

We also choose to incorporate the Panicek et al.^(3)^ proposal to include terms indicating benignity (“suggestive”) or malignancy (“suspicious”), which are appropriate at a cancer center. Although most of the participants supported the proposed initiative to create a standard lexicon, considerable proportions of the radiologists expressed partial receptivity or even disapproval of the proposal (28% and 4%, respectively). The proportion of resident physicians and fellows in radiology who were receptive to the proposed standardization (68%) was similar to that observed for the (more experienced) radiology specialists (69%). That particular finding stands in contrast to the fact that Panicek et al.^(3)^ found less overall acceptance among experienced radiologists. In addition, the receptivity of the majority of the referring physicians (86%) suggests enthusiasm for more objectivity in reporting by radiologists. Finally, it is important to point out that direct contact (in the department, via telephone call or in multidisciplinary meetings) should always be the alternative of choice to clarify any questions on the content of a report.

### Imaging descriptors from the TNM system

#### Brief description of vascular involvement

Most (94%) of the referring physicians reported that they preferred succinct descriptions of vascular involvement, compared with only 64% of the radiologists. That difference might be related to the consequences of the definitive establishment of vascular infiltration by imaging. Because the presentation of a disease on imaging can be deceptive, caution is required in assuming an emphatic position with major repercussions for therapeutic management. In addition, “external” impediments, such as suboptimal acquisition under limited clinical conditions, imaging artifacts, and the possibility of litigation prompted by a misdiagnosis, lead radiologists to prefer more descriptive profiles and a neutral stance.

#### Reporting of vessel wall irregularities

Most (78%) of the radiologists considered the reporting of vessel wall irregularities to be relevant, compared with only 47% of the referring physicians. That might reflect the lack of scientific evidence and uncertainty about whether this feature truly represents vascular involvement by tumors in most cases. Nevertheless, according to recent guidelines^(8)^, vessel wall irregularities constitute a well-established factor guiding the therapeutic approach to pancreatic cancer. In addition, irregularities in the contours of cervical vessels encased by an upper aerodigestive tract neoplasm pose a risk of imminent rupture (e.g., carotid blowout syndrome) and must be recognized promptly.

#### Reporting of the features of nodal capsule rupture

In contrast to what was found for the reporting of vessel wall irregularities, 92% of the referring physicians and 60% of the radiologists considered it relevant to report the features of nodal capsule rupture. Such rupture is of recognized importance in the prognostic stratification of patients with head and neck neoplasms, given that the spread of metastases substantially worsens survival. Radiologists working in other subspecialties might be less familiar with the related terminology or might rarely encounter the situation in their practice.

#### Reporting of lymph node numbers

Half (50%) of the referring physicians expressed a preference for precise numerical counts of lymph nodes appearing on imaging, whereas few (9%) of the radiologists reported providing such counts, 80% reporting that they refer to lymph node numbers in a more generic manner. In recent TNM system updates, the number of unusuallooking lymph nodes has increasingly been recognized as an isolated factor affecting patient survival rates, as seen for pancreatic and rectal cancer^(8)^. Therefore, radiologists specializing in oncologic imaging should be increasingly aware of this factor. In a disseminated lymph-node disease scenario, the use of generic expressions (e.g., “multiple”) or an established numerical reference (e.g., “more than/ at least a dozen”) might be a satisfactory compromise between radiologists and referring physicians.

Our study has some limitations. The relatively small number of participants limits the generalizability of the results, and the fact that the study was carried out at only one institution makes it vulnerable to selection bias. However, we believe that the study adds value by addressing a topic that is rarely studied in the specialized literature.

## CONCLUSION

Our assessments indicate that poor communication and differences in report interpretation between radiologists and referring physicians are still prevalent in radiology, as well as that oncology education in radiology usually focuses on the recognition of patterns and their associations with illness, less emphasis being placed on the descriptions of imaging findings and how those can be interpreted by referring physicians. It is therefore important to identify and understand the barriers and challenges to effective communication, searching for alternatives to avoid confounding factors. Given that the purpose of a radiology report is to convey information relevant to patient management, efforts to increase reporting objectivity and avoid confounding factors could improve the quality of care.
